# Brain responses to facial attractiveness induced by facial proportions: evidence from an fMRI study

**DOI:** 10.1038/srep35905

**Published:** 2016-10-25

**Authors:** Hui Shen, Desmond K. P. Chau, Jianpo Su, Ling-Li Zeng, Weixiong Jiang, Jufang He, Jintu Fan, Dewen Hu

**Affiliations:** 1 College of Mechatronics and Automation, National University of Defense Technology, Changsha, Hunan, China; 2 Institute of Textiles & Clothing, Polytechnic University of HongKong, Hung Hom, Kowloon, Hong Kong; 3 Department of Biology and Chemistry, City University of HongKong, Kowloon, Hong Kong; 4 Department of Fiber Science & Apparel Design, Cornell University, USA

## Abstract

Brain responses to facial attractiveness induced by facial proportions are investigated by using functional magnetic resonance imaging (fMRI), in 41 young adults (22 males and 19 females). The subjects underwent fMRI while they were presented with computer-generated, yet realistic face images, which had varying facial proportions, but the same neutral facial expression, baldhead and skin tone, as stimuli. Statistical parametric mapping with parametric modulation was used to explore the brain regions with the response modulated by facial attractiveness ratings (ARs). The results showed significant linear effects of the ARs in the caudate nucleus and the orbitofrontal cortex for all of the subjects, and a non-linear response profile in the right amygdala for only the male subjects. Furthermore, canonical correlation analysis was used to learn the most relevant facial ratios that were best correlated with facial attractiveness. A regression model on the fMRI-derived facial ratio components demonstrated a strong linear relationship between the visually assessed mean ARs and the predictive ARs. Overall, this study provided, for the first time, direct neurophysiologic evidence of the effects of facial ratios on facial attractiveness and suggested that there are notable gender differences in perceiving facial attractiveness as induced by facial proportions.

Facial attractiveness is a facial attribute that conveys significant biological advantages as expressed in mating success^1^, earning potential^2^ and longevity^3^, across different cultures and age groups^4^. Facial attractiveness judgment even exists in infancy^5^. A large body of studies have found that facial attributes that contribute to attractiveness include averageness^6^,^7^,^8^,^9^, symmetry^9^,^10^, sexual dimorphism^11^,^12^, expression^13^, and skin texture^14^. Among these factors, averageness and symmetry were found to be important criteria. Attractiveness increases with an increasing level of averageness and symmetry, which can be understood as evolutionary pressures that operate against the extremes of the population^15^.

Apart from averageness and symmetry, the sizes of individual features significantly influence the perception of facial attractiveness. Previous studies that address measuring certain facial features or manipulating individual feature sizes have found that female faces are more attractive when the faces have certain features, such as large eyes, prominent cheekbones, thick lips, thin eyebrows and a small nose and chin^16^,^17^. It is believed that an average face is attractive, but not optimally attractive, because attractive composite faces can be made to be more attractive by changing some of the feature sizes to be different from the sample mean^18^. Moreover, enhanced masculine facial characteristics increase both perceived dominance and negative attributes (e.g., coldness or dishonesty), which suggests that humans have a selection pressure that limits sexual dimorphism and encourages neoteny^11^.

In addition, some of the other behavioral studies have also attempted to make clear whether the facial measurements should follow certain defined ratios for attractive faces^19^,^20^. For example, men with higher width-to-height ratios (fWHRs) of faces are positively associated with having self-perceived power^21^, perceived dominance, and attractiveness to women for short-term relations^22^,^23^. A recent study reported that there possibly exist golden ratios of the face’s vertical distance between the eyes and the mouth and of the horizontal distance between the eyes, although different faces have varying attractiveness^24^. In particular, Fan *et al*.^25^ investigated the effect of facial proportions on facial attractiveness using computer generated face images with controlled skin tone and expression, and they found a different ideal facial proportion for facial attractiveness.

Despite the substantial number of behavioral studies on the influence of facial proportions or features sizes on facial attractiveness, little is known of how human brains respond to facial attractiveness as induced by varying the facial proportions. Past neuroimaging studies using real faces have found that some emotion- and reward-related regions, which involve the orbitofrontal cortex (OFC), anterior cingulate cortex, nucleus accumbens, caudate, and amygdala, respond to facial attractiveness^26^,^27^,^28^,^29^. More recent meta-analyses have observed consistent activations to be associated with facial attractiveness across neuroimaging studies^30^,^31^, especially in putative reward circuitry, such as linearly increased responses in the medial orbitofrontal cortex and nucleus accumbens for attractive faces^27^, and non-linear responses in the amygdala for both attractive and unattractive faces^26^. However, there are multiple dimensional aspects of facial characteristics that influence facial attractiveness perception, such as facial expressions, hairstyles and skin tones. Hence, it is extremely difficult to disentangle the effect of an individual factor from the composite effects of these factors in natural face images, despite a recent trend in exploring the high-dimensional nature of neural representations that characterize social perception in natural and realistic environments^32^. In the present study, we therefore used computer-generated, yet realistic face images that had varying facial proportions as stimuli to investigate the brain responses to facial attractiveness as induced by facial proportions. This approach allowed us to explore only the effect of facial ratios on facial attractiveness using an event-related fMRI design, by manipulating the individual facial ratios and controlling the effects of other confounding factors, such as the hairstyle, skin texture and expression, to test whether some of the facial ratios or their combinations contribute to facial attractiveness.

Another objective of this study is to investigate whether there is any difference in the brain responses to facial attractiveness as induced by facial proportions between male and female viewers. Behavioral studies have indicated that men assign greater importance to physical attractiveness compared with women when evaluating a potential mate^33^. More specifically, sexual preferences for some facial features, such as lip size, mouth width, cheekbones height and chin size, could result in gender differences in the perception of facial attractiveness^20^,^34^. Furthermore, our recent behavioral studies have demonstrated gender differences in ranking facial physical attractiveness^25^. In fact, there has been some evidence of subject gender-by-attractiveness in judging face attractiveness, especially in evaluating opposite-sex faces^26^,^33^,^35^. For example, increased responses in some reward-encoding regions such as OFC^35^ and anterior cingulate^26^ were observed only for male participants. When viewing male faces, women showed stronger linear effects, while men showed stronger nonlinear effects, etc. These findings suggest that the reward value of facial attractiveness is more pronounce in men than that in women, and support sex differences in mate selection in that men identify attractiveness as a stronger motivation^35^. Inspired by these previous findings, we therefore speculate that there exist gender differences between the neural responses of male and female brains, which underlie the behavioral discrepancy between genders in the judgment of facial geometric attractiveness.

## Methods

### Face images as stimuli

**Figure 1:**
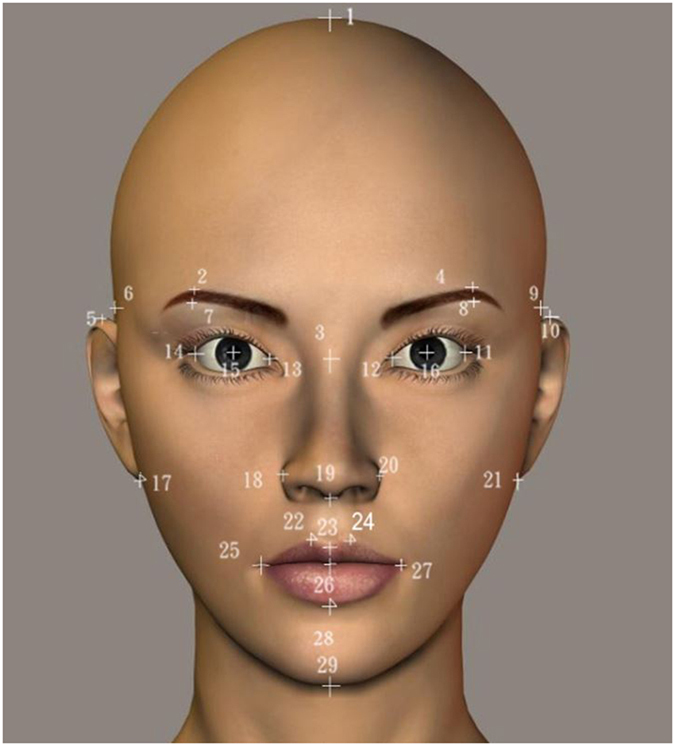
The “Original Face” and a set of 29 facial landmarks used in the geometric morphometrics analysis.

**Figure 2:**
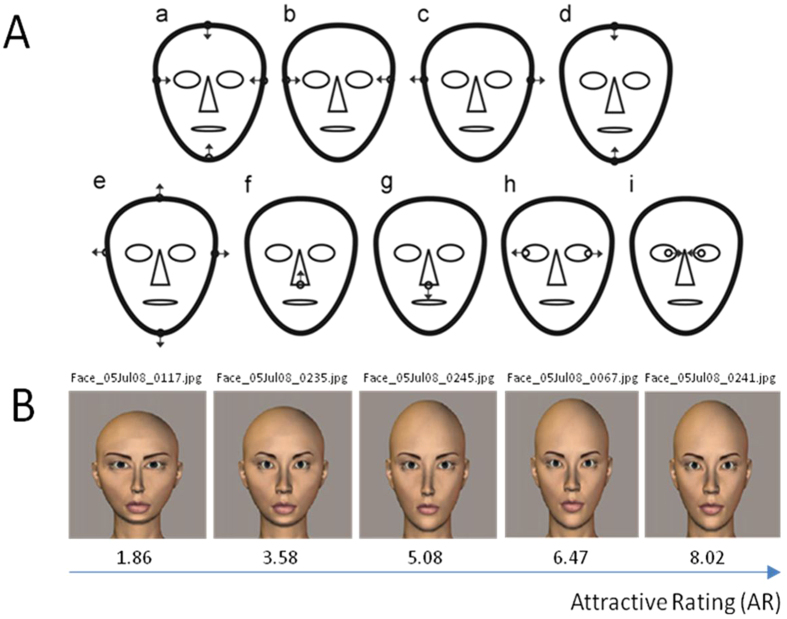
The alterations of the “Original Face” to generate the other facial images. (**A**) demonstrates nine different approaches (a–i) of changing the facial ratios (refer to Fan *et al*.,^25^ for detail). (**B**) Examples of facial images generated for this study.

All of the face images that were used as stimuli in this study were selected from our previous study^25^. All of them were created by altering certain key facial dimensions using computer software from a so called “Original Face” (See Fig. 1), which was obtained by averaging the features of the facial images of some arbitrarily chosen famous oriental ladies (Japanese, Korean and Chinese) that were available on the internet. Furthermore, the faces were hairless to eliminate the effects of hairstyles, and they were controlled to have the same neutral facial expression and skin tone. Finally, we created sufficient but realistic variations in the facial dimensions and ratios of the facial images, by applying gradual alteration to the “Original Face” with nine different approaches (See Fig. 2 and also refer to ref. 25 for details of the method). During each approaches, the maximum possible alteration of the targeted dimension(s) without being overly unrealistic was determined by a preliminary visual assessment. Then, the change in each step of the alterations was calculated by dividing the maximum possible dimensional alternation by 14. This strategy resulted in 14 progressive facial images in each approach. The above nine approaches created wide variations in the length and width of the face, the length of the nose, and the positions of the eyes, while keeping other features such as the size of the eyes, the width of the nose, and the size and shape of the mouth unchanged. Furthermore, based on the “original face” and the selected 18 facial images created by the above 9 approaches, the mouth width, eye fissure width, nose width and lip height were altered in a pre-defined proportion by the orthogonal experimental design^25^. Using this method, a total of 432 face images were created for further study.

The above approach of manipulating individual feature sizes provides a simple but efficient way in which we can search the entire space of face shapes starting from the mean face of a cohort, with minimization of the sample size and preservation of the symmetry. Unlike previous studies that intentionally exaggerate masculine or feminine facial characteristics^11^, variations in the facial proportions or feature sizes were created by a stepwise procedure (e.g., −10%, −5%, + 5% and  + 10% for the mouth width), regardless of the sexual dimorphism dimension. Moreover, the levels of proportional changes for all of the facial ratios were optimally determined, to have a wider range of variation but without creating too unrealistic images. These strategies for face image generation facilitate the investigation of only the effect of facial ratios on facial attractiveness as independent variables.

All of the face images have variable geometric morphometric parameters that consist of morphological and/or functional points as well as contours of the eyes, nose and mouth. Based on the previous literature^36^, we further identified 29 landmarks (See Fig. 1) for each sample image using computer software, and we generated the 21 ratios listed in Table 1 by measuring the vertical or horizontal distances between these landmarks. The measures of the 21 ratios compose the low-level features that result in facial attractiveness for an individual face image. Furthermore, for each face, its distance from the ‘original face’ was calculated by the Euclidean distance between its ratio features and the ratio features of the ‘original face’.

### Human Subjects

fMRI data were collected from 41 healthy subjects (22 males) with normal or corrected-to-normal vision who gave written informed consent to participate in this study. All of the subjects were revealed to have non-homosexual preferences by using a debriefing questionnaire. There was no significant difference between the ages of the males and females (age: 23.57 ± 1.10, *p* = 0.745). All of the participants were in good health and had no past history of psychiatric or neurological diseases. Only 36 subjects (19 males) were available for further analysis after head motion inspection (see the Data Preprocessing section). This study was conducted according to the principles in the Declaration of Helsinki and approved by the Ethics Committee of the Third Xiangya Hospital of Central South University, Changsha, China.

### Experimental paradigm

Each human subject underwent an explicit judgment task of facial attractiveness on the face stimulus. Face images (800 × 600 pixels; subtending 23 × 18° of visual angle) were presented in the center of the SVGA display, using an MR-compatible liquid crystal display goggle operating at a resolution of 800 × 600 pixels, at 60 Hz. The stimuli series were generated with the E-Prime software (http://www.pstnet.com/eprime.cfm). The subjects were scanned in four runs, and each run involved 108 face presentation trials in which a face image was presented for 1 second followed by a fixation cross for a further 2 seconds, and the subjects were instructed to maintain fixation. In each run, 54 null event trials, in which a fixation cross was presented for 3 seconds, were randomly interspersed with the face presentation trials. These face images (432 faces in total) were selected once for all of the subjects, and were randomly presented during the scanning. The scanner was in the acquisition mode for 15 seconds before each series to achieve steady-state transverse magnetization. Following the presentation of each face, the subjects were instructed to judge the attractiveness by pressing one of four buttons with their right hands (denoting “highly attractive” and “attractive”) and their left hands (denoting “unattractive” and “highly unattractive”) on a response keypad, respectively. Their response time from the presentation of a face to the pressing of a button was also recorded.

### fMRI data

Data were collected on a 3.0T MRI scanner (Philips, MR Ingenia, the Netherland) at the Third Xiangya Hospital of Central South University, Hunan Province, China. To reduce their head motion, the participants’ heads were fixed using foam pads with a standard birdcage head coil. The functional images were collected using a gradient-echo T2*-weight echo-planar imaging (EPI) sequence with the following parameters: repetition time (TR) = 3000 ms, echo time (TE) = 40 ms, field of view (FOV) = 240 × 240 mm, flip angle (FA) = 90°, slice thickness = 2 mm, gap = 1 mm, and matrix = 88 × 86. The volumes consisted of 36 slices angled at −30° to the horizontal, which could improve the signal quality in the ventral prefrontal cortex and amygdala^26^. Each session lasted 8 min and 15 seconds, and 165 volumes were obtained. Finally, T1-weighted anatomical images were acquired for each subject for detailed anatomical information.

### Debriefing

The participants undertook one debriefing task outside the scanner immediately after the experiments. They were asked to rate all of the faces in term of attractiveness, using a computerized visual analogue scale. The scale was marked with the extremes of “highly unattractive” and “highly attractive”, with the mid-point marked. The ratings were scaled between 1 and 9, with 9 representing ratings of the highest attractiveness. The mean attractiveness rating (AR) for each image was obtained by averaging the ARs over all of the subjects.

### Data preprocessing

Imaging data were preprocessed and analyzed using SPM8 (Wellcome Department of Imaging Neuroscience, London; http://www.fil.ion.ucl.ac.uk/spm). For each participant, the first three volumes of scanned data were discarded because of magnetic saturation effects. The remaining volumes were realigned to the first image for correcting the head motion, and a mean functional image was correspondingly obtained. Three male and two female participants whose head motion exceeded 1.8 mm were discarded, and the residual 36 participants had less than 1 mm of translation in the *x*, *y*, or *z* axis and 2° of rotation in each axis. The images were then normalized to an MNI echo planar imaging template with an affine registration followed by a nonlinear transformation, using a voxel size of 3 × 3 × 3 mm. Finally, a 6-mm FWHM Gaussian kernel was used to smooth the data.

### Statistical parametric mapping analysis

The functional images were analyzed statistically using a two-stage procedure to explore the brain responses while judging the attractiveness of faces that had varying facial proportions. In the first stage, we estimated the effect sizes for each regressor in the design matrix within the framework of the generalized linear model, with parameter modulation for each subject. To identify the brain regions that showed a linear or nonlinear effect of attractiveness ratings, we used a parametric approach^37^ that allows one to characterize brain responses as a linear combination of (basis) functions of the experimental parameter (e.g., *f*(*x*) = *a* + *bx* + *cx*^2^, where *x* is the subject-specific attractiveness ratings). We used a first-order and a second-order polynomial expansion, respectively, as the basis functions in which each polynomial term represents an interaction between the attractiveness ratings and a boxcar stimulus function *box*. The standard boxcar function (i.e., zeroth order) modes the difference between the face presentation and the silent baseline condition, irrespective of the face attractiveness. The linear (i.e., lin = *r · box*) term and the quadratic (i.e., *se*c = *r*^2^* · box*) term account for the linear and second-order nonlinear changes in the blood oxygen level dependent (BOLD) signal relative to the parameter of facial attractiveness ratings, respectively. Prior to model fitting, these terms (as parametric modulations in SPM8) were convolved with a hemodynamic response function.

In the design matrix of the first level model, the covariates of interest include the linear term and the quadratic term, both of which model all of the trials on one regressor but includes a linear or second-order nonlinear parametric modulation of this regressor by using subject-specific ratings for each image. A linear (*f(x)* = *a* + *b* · *AR*) and a quadratic nonlinear polynomial function (*f(x)* = *a* + *b* · *AR* + *c* · *AR*^2^) of attractiveness ratings (ARs) were used to estimate the regions that had linear and nonlinear effects of facial attractiveness, respectively. In the linear expansion model, the remaining columns (confounds or covariates of no interest) include the zero*th*-order term (modeling the main effect of face presentation irrespective of attractiveness), a constant term, regressors that pertain to the reaction time for each trial and six head movement parameters. However, in the quadratic expansion model, the linear term of attractiveness ratings was also moved into the covariates of no interest. The obtained effects of the linear term and the second-order term across the population of subjects were submitted to the second stage of analysis, in which the significance of the linear and nonlinear effects was tested by using random effects analyses with ANOVA models in SPM8.

In the second level analysis, men and women were treated as a single group, and a one-sample *t*-test was used to evaluate the linear or second-order nonlinear effects of the facial attractiveness irrespective of the subject gender. Additionally, for estimating the effect of the subject gender-by-attractiveness, the males and females were also treated as separate groups and a two-sample *t*-test (with contrasts of 1 for the males and −1 for the females) was applied to investigate the gender difference in the brain responses to facial attractiveness. All of the activation was reported by using the more liberal threshold of *p* < 0.001 (uncorrected) to explore potential regions that respond to perceiving the attractive faces. We also used small volume corrections (SVCs, 12 mm radius) with *p* < 0.05 as the significance threshold to indicate whether the activation within the regions of interest (ROIs) can survive for multiple comparisons over small regions of interest. To show the modulation effect of the attractiveness ratings on the brain responses for each subject, we also computed the average BOLD signals for all of the images within an individual attractiveness group as defined by the button that the subject pressed. A bar figure was used to indicate the relative percent signal change of the BOLD signal for different attractiveness-level face images.

### Learning the most relevant face ratios from the fMRI responses

Next, we attempted to identify the most relevant low-level feature (face ratio) sets with facial attractiveness, based on the fMRI scans. First, we constructed the fMRI-derived semantic feature sets that were relevant to facial attractiveness, which comprise the neural response of all of the ROIs that significantly linearly responded to the facial attractiveness ratings. The data were then normalized to represent percentage signal changes with respect to the temporal mean of each run. The brain volumes that corresponded to each image trial were extracted from the time series with a lag of 4 s to account for the delay in the hemodynamic response. Using the statistical parameter model, we identified eight brain ROIs that had linear effects of facial attractiveness, including bilateral OFC, caudate, mOFC and postcentral gyrus (See the Results section and Table 2). The signals at the peak within the ROIs were concatenated for the representation of fMRI-derived high-level semantic features that were relevant to the face attractiveness.

Second, we performed canonical correlation analysis (CCA) to extract the most correlated variables from the fMRI-derived semantic features sets and the facial ratio feature sets. CCA can obtain the correlation structure of two sets of latent variables, which represent a set of independent variables and a set of dependent variables^38^. Assuming two sets of variables, which are denoted


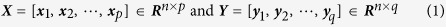


CCA is used to extract the correlated modes between vectors ***X*** and ***Y***, by seeking a set of transformation vector pairs, ***A***_i_ ∈ ***R***^*P*×1^ and ***B***_i_ ∈ ***B***^***q***×1^, in such a way that the linear correlation of the canonical variates ***u***_i_ ∈ ***R***^*n*×1^ and ***v***_**i**_ ∈ ***R***^*n*×1^ is maximized:


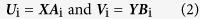


where symbol *i* denotes the *i*-th pair of transformation vectors, ***A***_i_ and ***B***_**i**_, which result in the *i*-th pair of variates ***U***_i_ and ***V***_i_. The correlation between ***U***_i_ and ***V***_i_ is


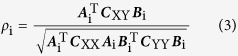


where ***C***_XY_ is the cross-covariance matrix of ***X*** and ***Y***, while ***C***_XX_ and ***C***_XY_ are auto-covariance matrix. By solving the maximization problem of Eq. (3), we obtain the ascending ordered correlation values {*ρ*_1_, *ρ*_2,_ …,*ρ*_*r*_} with *r* ≤ min(*p*, *q*), and the corresponding transformation matrices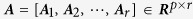 and 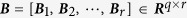, in such a way that the elements within 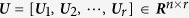 and 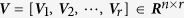 are orthogonal, and the correlation between ***U***_i_ and ***V***_i_ is maximized. Thus, the predictability between ***U***_i_ and ***V***_i_ is maximized.

Here, we considered ***X*** to be the fMRI-derived high-level semantic feature vector (the averaged response across all of the subjects to each face image) and ***Y*** to be the low-level feature vector (the facial ratios of each face image). With CCA, we learned the most relevant components of the face ratios to the face attractiveness, and the corresponding transform model is represented by the transform matrix ***B***. Note that all of the input data were Z-score normalized prior to the CCA.

### Predicting facial attractiveness based on fMRI-derived face ratios

We obtained six components of the face ratios that are the most relevant to face attractiveness via the CCA (see the Results for details). Next, we used the following transformed Gaussian formula to capture the main trend in each of the six components:


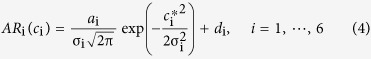


where 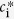 is the transformed value with the Box-Cox transformation function


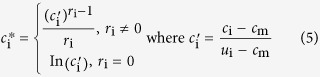


where *c*_m_ is an arbitrary value that is chosen to ensure that 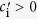. The values of *a*_i_, *d*_i_, *u*_i_, *σ*_i_ and *r*_i_ can be determined by applying nonlinear regression.

We constructed the following prediction model of the attractiveness ratings by assuming that the overall facial attractiveness is the linear combination of the contributions of each of the six components:


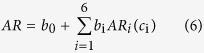


where the coefficients *b*_0_ and *b*_*i*_(*i* = 1, …, 6) can be obtained by multiple linear regression based on the least-squares model.

## Results

### Linear effect of attractiveness ratings

**Figure 3:**
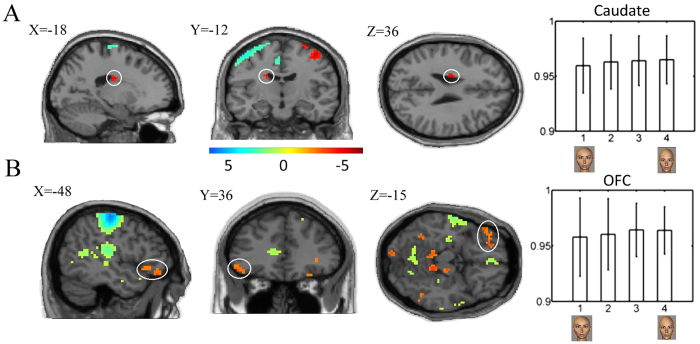
Linear effects of the attractiveness ratings for all of the subjects. (**A**) demonstrates the linear effect of the attractiveness ratings in the caudate nucleus (left panel) and the relative change in the BOLD signals across four different groups (right panel), with group 1 representing the highly unattractive faces and 4 denoting the highly attractive faces. (**B**) The orbitofrontal cortex (OFC) shows significant positive linear effects of attractiveness ratings (left panel). In other words, more attractive faces evoke stronger activation in the OFC (right panel). Here, *p* < 0.001 uncorrected is used as the threshold for display. The stimuli were divided into four groups for each subject based upon which button was pressed. Then, the BOLD responses are averaged for each cohort of faces. Because there was no baseline condition in the experimental design, only the relative values of the response with respect to the mean activity over each subject were calculated. The units are the % signal change, and the error bars represent the standard error of the mean over the subjects.

We used a linear term of the mean facial attractiveness ratings with a one-sample *t*-test to evaluate the linear changes in the BOLD signal relative to the facial attractiveness. The results are listed in Table 2. Figure 3 shows a significant positive linear effect of the average attractiveness ratings (higher attractiveness ratings associated with linear increases in the BOLD signals) with the significance level of *p* < 0.05 (SVC; cluster size >5 voxels) in the caudate nucleus (Fig. 3A), inferior frontal gyrus (Fig. 3B) and right postcentral gyrus, as well as a significant negative linear effect in the left postcentral gyrus.

### Non-linear effects of attractiveness ratings

**Figure 4:**
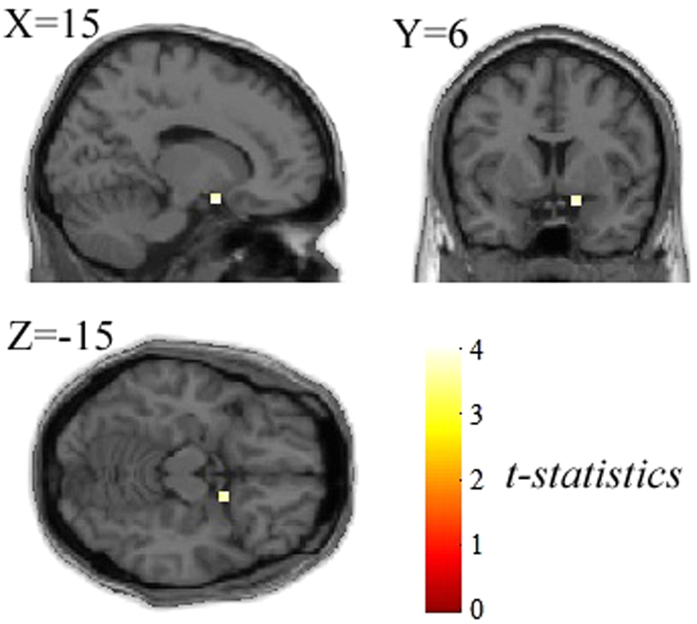
Demonstration of the differential nonlinear effects of the attractiveness ratings within the right amygdala between the male and female observers (males vs. females, *p* < 0.05, SVC).

A second-order term in the polynomial expansion of facial ARs was used to test whether the addition of a quadratic term accounts for the change in the BOLD signal relative to the subject-specific attractiveness ratings. Only males showed significant nonlinear effects in the right superior frontal gyrus, right superior parietal gyrus, right angular gyrus (BA40) and right precuneus (BA7) (see Table 3 for details). The direct comparison between the males and females demonstrated the significant interaction effects between subject gender and attractiveness ratings in the left cuneus, superior occipital gyrus, angular gyrus, and right amygdala (*p* < 0.015, SVC), as listed in Table 4. Notably, a significant positive second-order relationship between the BOLD activation and the facial attractiveness was observed in the right amygdala (Fig. 4).

### Decoding attractiveness ratings from responses to facial images

We considered the predictor *X* to be the fMRI-derived feature vector that consists of the BOLD responses within the eight ROIs and the predictant *Y* to be the 21 facial ratios. Next, we used CCA to extract the most relevant components of the facial ratios with respect to the facial attractiveness. We obtained eight components of the facial ratios that are represented by the columns in the transformation matrix ***B***. Furthermore, the two columns were removed due to their low correlation values (*p* > 0.001). The residual six columns represent the most relevant components of facial ratios to facial attractiveness, which are listed in Table 5. From the correlations between the six components and the ratios, it can be learned that some of the ratios have higher weights in determining the facial attractiveness. For example, the first and sixth components are largely related to the nose width and the interocular distance. The second and fourth components are largely related to the ratios of mideye distance and the interocular distance to the nose width, and so on. In particular, multiple components that involve the components 1, 3, 5 and 6 indicate that the ratio of the ear length to the interocular distance and the ratio of the lips-chin distance to the interocular distance play important roles in factors that influence the facial attractiveness, which is largely in line with the results from behavioral experiments^25^. Overall, the ear length, nose size, interocular distance and lip-chin distance are of more importance in determining the facial attractiveness compared with the other feature sizes. We have also visualized the effects of these components by averaging the 20 faces that have the highest and lowest scores on them (see supplemental Fig. S1).

**Figure 5:**
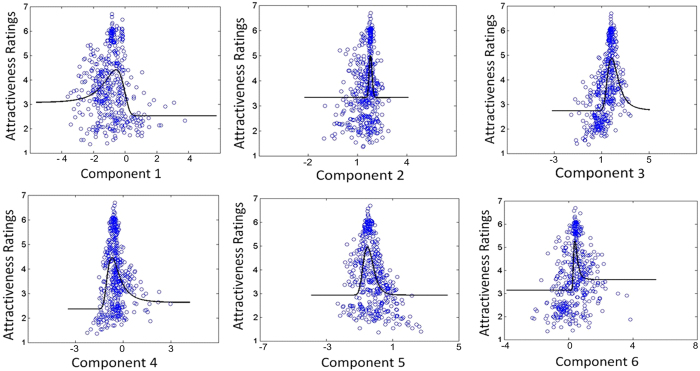
Demonstration of the attractiveness ratings as a function of the six components.

**Figure 6:**
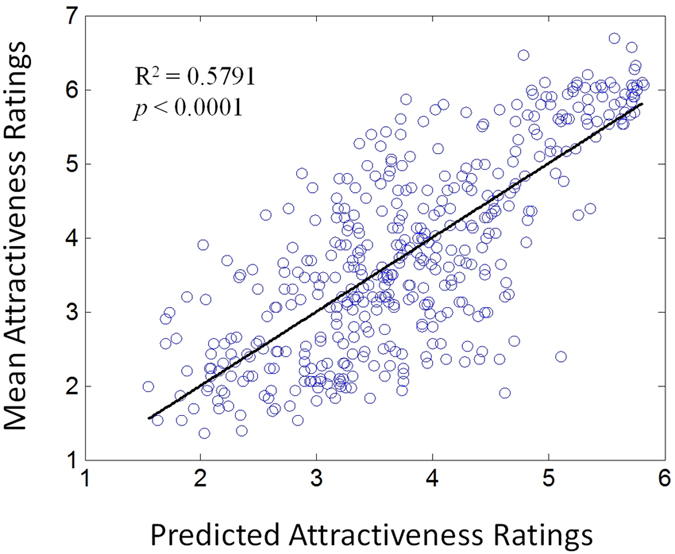
Significant correlation between the mean attractiveness ratings and the predictive attractiveness ratings for all of the face images.

Each component is plotted against the attractiveness ratings in Fig. 5. By applying nonlinear regression, we obtained the coefficients of model (4) and listed them in Table 6. Figure 6 plots the mean attractiveness ratings of the 432 facial images against the predicted attractiveness ratings using a regression equation (6). It is evident that the predicted attractiveness ratings are significantly linearly related with the visually assessed mean attractiveness ratings (*R*^2^ = 0.58, *p* < 0.001).

## Discussion

The present study aims to explore how human brains perceive the attractiveness of faces that have varying facial proportions while excluding the influence of facial expression, the preference of hairstyle and the skin tone. The results revealed that distributed cortical regions, including the caudate nucleus, OFC, and the amygdala responded to facial attractiveness as induced by variant proportions. Furthermore, the correlation analysis between the brain response intensity that was measured by fMRI and the low-level features of the facial ratios showed that there were obvious couplings between fMRI-derived semantics of facial attractiveness and specific components of facial ratios. These findings provide direct neurophysiologic evidence for the suggestion that facial proportions significantly influence the perception of facial attractiveness. Furthermore, activities in the amygdala exhibited significant interaction effects between the viewers’ genders and attractiveness ratings, which suggests a noticeable discrepancy between the male and female observers on perceiving the attractiveness of faces with morphological variations.

### Regions with responses to facial attractiveness

Our results show significant linear effects of facial attractiveness in the postcentral gyrus, caudate nucleus, and bilateral inferior frontal gyrus. Furthermore, the precuneus, angular gyrus, and amygdala exhibit significant non-linear effects of the facial ARs for the males. These observations suggest that there exist distributed regions that are involved in evaluating the attractiveness of face images with variant proportions, even when eliminating the influence of facial expression, hairstyle and skin tone.

We observed a significant linear effect of attractiveness ratings in the caudate nucleus. It has been previously reported that the caudate nucleus was sensitive to the emotional content of stimulation, including the viewing of a loved romantic partner^39^, and the experience of beauty^40^,^41^. Furthermore, Bartels and Zeki have reported that the caudate nucleus and putamen are linked to both positive and negative emotion. Notably, the reported location where the caudate nucleus was activated is similar to the location of activity observed in previous studies about beauty and romantic love^42^. This evidence supports my suggestion that the appropriate ratios of faces induce a facial attractiveness experience, to evoke the activation of the caudate nucleus, which could be parametrically modulated by the strength of the experience. More evidence on the response of the caudate nucleus to facial attractiveness has been reported recently^31^,^43^.

Another key region that demonstrates significant linear effects of attractiveness ratings is the posterior orbitofrontal cortex (inferior frontal cortex). It has been observed that the orbitofrontal cortex (OFC) is involved in reward and has complex response patterns in the perception of attractive faces^44^. It have been observed that the medial and lateral OFC respond to facial attractiveness and unattractiveness, respectively^28^, which suggests that rewards and punishments are represented separately in the OFC. However, this response pattern was not replicated in another experiment^26^, which instead exhibited significant non-linear responses to attractiveness in a portion of the medial OFC. Our results showed no response to attractiveness in the medial OFC. In the lateral OFC, however, we observed more responses to high attractive faces than to low attractive faces. This opposite response pattern compared with the previous report in which the lateral OFC showed greater activation to unattractive relative to attractive faces^28^ is difficult to interpret, although other types of reward such as pleasant gustatory stimuli have been found to activate the lateral OFC^45^. One possible explanation is the use of realistic faces with varying expressions as stimuli in O’Doherty *et al*.^28^ which could introduce more dimensions of perceiving the facial attractiveness, such as sexually dimorphic facial features and emotions. These controversial results also suggest the OFC is involved in rewards in a rather complex manner.

### Subject gender matters in the perception of facial proportions

As expected, we observed a positive non-linear response profile of facial attractiveness ratings in the right amygdala, which is in line with the previous finding of increased neural activity in the amygdala to both extremely attractive and extremely unattractive faces^26^,^31^, which supports the role of the amygdala in social and emotional perception. More importantly, the activation in the amygdala showed a significant gender-by-attractiveness interaction (Fig. 4). In other words, the males exhibited greater responses to highly attractive and highly unattractive faces compared with middle-rank faces in the amygdala, whereas the effect in the females fails to pass the test for statistical significance.

To our knowledge, there is no evidence that shows that only opposite-sex faces lead to nonlinear responses in the amygdala. In fact, women show a non-linear response in the right amygdala when viewing male or female faces, as do men^26^. For both men and women, however, looking at opposite-sex faces produces stronger activity in the amygdala than looking at same-sex faces at the both ends of the attractiveness continuum, although the difference between the subjects and face genders has not achieved the statistical significance level^26^. Nevertheless, a clear difference in how men and women rate female facial attractiveness has been observed^46^. Moreover, attractive female faces activate reward regions in men more than attractive males or unattractive faces of either gender^27^. In this paper, the original face was obtained by averaging the faces of a cohort of young women. This approach makes most of the facial images appear to be more feminine. Hence, we speculate that the subject-gender differences in the activation of the amygdala are likely due to an opposite-sex bias in facial attractiveness evaluation. In other words, for heterosexual individuals, opposite-sex faces could hold greater significance or reward value than same-sex faces^33^, which is supported by consistent evidence for opposite-sex biased neural responses in the amygdala^27^,^47^, OFC^48^ and additional reward-related structures such as the nucleus accumbens^49^ and ventral tegmental area^27^.

Furthermore, the observed gender differences in the activation of the amygdala suggest differential preference to facial proportions between the subject genders. Given that the response in the amygdala could code the emotional value^26^ and track the stimulus intensity^31^, the finding that the male subjects showed a more significant nonlinear response in the right amygdala than the female subjects, implies that men are likely to assign more importance than women to facial proportions or shape in the facial attractiveness experience. This finding is also supported by the behavior and neuroimaging evidence that compared with women, men would pay more attention to facial physical attractiveness in mate preferences^33^,^35^.

### Attractive faces have ideal facial measurements

In this paper, we found that facial attractiveness induced by facial proportions evoked a neural response within some of the reward regions. Furthermore, the significant negative correlation between the attractiveness ratings and the distance of the face images away from the ‘original face’ (average face) was observed (see supplemental Fig. S2), i.e., a face was observed to be more attractive when its ratios were close to the average. This finding suggests that the observed modulated effects within some specific regions in the reward circuit are likely attributed to composite effects of facial ratios on the averageness of the facial shape and some of the sexual dimorphic features, which lead to a distinct perception of facial attractiveness. Indeed, a quantitative analysis of female faces by manipulating individual facial features has demonstrated that facial attractiveness benefitted from averageness either at a overall level (e.g., the face shape), or for many individual facial features (e.g., eyes, nose, mouth)^16^. Moreover, some of the sexually dimorphic features are not of average size, and in the contrast, were larger or smaller, which would enhance attractiveness^16^,^18^. For example, feminine traits such as small jaw and full lips for female faces and masculine traits such as thick brow ridges and a large jaw structure for male faces are suggested to enhance the ratings of facial attractiveness by opposite-sex subjects^24^,^36^.

Given that these responses can be modulated by subject-specific attractiveness ratings, we sought to identify the most relevant components of the facial ratios to facial attractiveness by correlating the low-level facial ratio features and the intensity of the neural activity. Based on these components, we learned a regression model that can effectively predict the mean attractiveness ratings of each face image. This approach suggested that the neural activity in the activated regions contains meaningful high-level semantic information about facial attractiveness, which further confirms the suggestion that facial ratios or proportions contribute to facial attractiveness. Similar methods have been applied to predicting the semantics of nouns that are represented by fMRI neural activation^50^ and bridging the low-level features of video stimuli and fMRI-derived high-level semantics for video classification^51^.

Our results also suggested that attractive faces have optimal facial ratios or proportions. The previous literature has reported that beautiful faces have facial measurements that are close to the golden ratio^36^. These studies, however, used realistic face images as experimental materials that are difficult to control the effects of variations in hairstyles, skin texture, skin color and facial expressions. The current study investigates only the effect of the facial ratios, by excluding the impact of these confounding factors. Our results indicate that some specific facial ratios play important roles in determining the facial attractiveness. This finding provides us additional neurophysiological evidence for the suggestion that attractive faces follow optimum facial proportions.

### Limitations

Here, we used computer-generated artificial face images as stimuli instead of images of realistic human faces. However, it should be noted that the mechanism of viewing a synthetic face is somewhat different from that of viewing a real face. In some face-sensitive structures such as the fusiform gyrus, the response to human facial expressions of emotions is significantly stronger than that of viewing computer-generated faces^52^. Hence, a further validation on real faces might be necessary, although in such a study, it is a large challenge, if not impossible, to control the facial expression, hairstyle, and skin tone and texture in real face images. Another feasible avenue for addressing this issue is to utilize the data-driven approach to explore the high-dimensional nature of face beauty based on realistic face images. The data-driven approach does not manipulate the features and need not constrain some of the variants that are of no interest. However, due to the high-dimensional traits of the facial attractiveness space (e.g., sexual dimorphism, symmetry, averageness, expression, hairstyle, shape, skin tone), a large dataset and powerful data-driven algorithm are required to tackle a truly ‘holistic’ outcome of face attractiveness perception, in which different characteristics or dimensions interact in more complicated ways.

## Conclusions

To conclude, the present study indicated that some specific brain regions, including the caudate nucleus, OFC, and amygdala, were involved in the perception of attractiveness of faces that have varying facial proportions. Furthermore, we found notable between-gender differences in the neural activity in the right amygdala, which suggests that there is a discrepancy between the females and males in the brain responses to faces that have different proportions. To our knowledge, these observations for the first time provided neurophysiologic evidence for the hypothesis that human faces with variable proportions have differential attractiveness, even excluding the influence of hairstyle and facial expression as well as skin tone and texture.

## Additional Information

**How to cite this article**: Shen, H. *et al*. Brain responses to facial attractiveness induced by facial proportions: evidence from an fMRI study. *Sci. Rep.* **6**, 35905; doi: 10.1038/srep35905 (2016).

## Supplementary Material

Supplementary Information

## Figures and Tables

**Table 1:** Definition of facial ratios.

Ratio number	Numerator points	Denominator points	Description
1	*y*10–*y*21	*x*12–*x*13	Ear length to interocular distance
2	*y*10–*y*21	*x*18–*x*20	Ear length to nose width
3	*x*15–*x16*	*x*12–*x*13	Mideye distance to interocular distance
4	*x*15–*x*16	*x*18–*x*20	Mideye distance to nose width
5	*x*25–*x*27	*x*12–*x*13	Mouth width to interocular distance
6	*x*23–*x*29	*x*12–*x*1*3*	Lips-chin distance to interocular distance
7	*x*23–*x*29	*x*18–*x*20	Lips-chin distance to nose width
8	*x*12–*x*13	*x*12–*x*11	Interocular distance to eye fissure width
9	*x*12–*x*13	*y*23–*y*28	Interocular distance to lip height
10	*x*18–*x*20	*x*12–*x*11	Nose width to eye fissure width
11	*x*18–*x*20	*y*23–*y*28	Nose width to lip height
13	*y*23–*y*28	*y*19–*y*26	Lip height to nose-mouth distance
14	*y*1–*y*29	*x*6–*x*9	Length of face to width of face
15	*y*19–*y*29	*y*26–*y*29	Nose-chin distance to lips-chin distance
16	*x*18–*x*20	*y*19–*y*26	Nose width to nose-mouth distance
17	*x*25–*x*27	*x*18–*x*20	Mouth width to nose width
18	*y*3–*y*19	*y*19–*y*29	Length of nose to nose-chin distance
19	*y*3–*y*19	*y*10–*y*21	Length of nose to length of ear
20	*x*12–*x*13	*x*18–*x*20	Interocular distance to nose width
21	*x*6–*x*9	(*x*18–*x*20)*4	Length of face to 4 times nose width

**Table 2:** Brain regions that show significant linear effects of mean attractiveness ratings; One-sample *t*-test, *p* < 0.05, SVC, extent threshold of five voxels.

Regions	BA	Hemisphere	MNI coordinate			Significance (*t*_*35*_)	Cluster size (voxels)
			*x*	*y*	*z*		
Postcentral Gyrus	4	Left	−33	−27	51	−6.12	389
		Right	36	−30	66	6.02	464
Rolandic Operculum		Right	51	−21	18	5.22	28
Superior Parietal Gyrus	7	Right	18	−75	51	3.90	8
Caudate		Left	−18	−15	24	3.71	29
		Right	15	−9	21	1.56	7
Inferior Frontal Gyrus		Left	−48	33	−15	2.35	33
			−21	45	−18	2.03	10
Inferior Frontal Gyrus		Right	24	12	−24	2.77	5
			27	36	−21	2.09	8

**Table 3:** Brain regions that show significant nonlinear effects of mean attractiveness ratings; One-sample *t*-test, *p* < 0.001, uncorrected, extent threshold of five voxels (male subjects).

Regions	BA	Hemisphere	MNI coordinate			Significance (*t*_*35*_)	Cluster size (voxels)
			*x*	*y*	*z*		
Superior Frontal gyrus		Right	30	0	69	5.20	127
Superior Parietal gyrus		Right	24	−69	60	4.31	10
Angular gyrus	40	Right	45	−63	48	3.68	20
Precuneus	7	Right	12	−57	69	3.59	8

**Table 4:** Brain regions that show significant gender-by-attractiveness interaction (the male group vs. the female group) in nonlinear effects of mean attractiveness ratings; Two-sample *t*-test, *p* < 0.015, SVC, or p < 0.00013, uncorrected, extent threshold of five voxels.

Regions	BA	Hemisphere	MNI coordinate			Significance (*t*_*35*_)	Cluster size (voxels)
			*x*	*y*	*z*		
Cuneus	19	Left	−6	−81	36	4.31	27
Superior occipital gyrus		Right	33	−75	42	4.23	18
Angular gyrus	40	Right	42	−60	45	4.15	32
Amygdala		Right	18	3	−18	3.89	11

**Table 5:** CCA transformation matrix for the facial attractiveness ratings.

Ratios	Components					
	1	2	3	4	5	6
1	**0.7345**	0.0926	**0.6444**	−0.1354	**0.4403**	**0.6838**
2	−**0.3325**	−0.0105	−0.2548	0.1009	−0.1742	−**0.3167**
3	0.0659	0.1402	0.1148	0.1362	−0.0688	0.0698
4	−0.0756	−**0.2328**	−0.0991	−**0.1922**	0.0914	−0.0878
5	−0.2397	−0.1973	−0.2531	−0.1144	−0.0981	−0.2356
6	−**0.4615**	−0.0207	−**0.4067**	0.1154	−**0.2222**	−**0.4259**
7	0.2366	0.0537	0.1564	−0.0597	0.0365	0.2381
8	−0.1145	−0.1355	−0.1194	−0.0881	0.0387	−0.1098
9	0.1210	−0.0076	0.1498	−0.0202	0.0299	0.0998
10	0.0727	0.0656	0.0621	0.0359	0.0138	0.0662
11	−0.2223	−0.0582	−0.2408	−0.0109	−0.1354	−0.1875
12	−0.0109	0.0025	−0.0292	−0.0039	0.0170	−0.0117
13	−0.0875	−0.0724	−0.0735	−0.0342	−0.0728	−0.0794
14	0.0055	−0.0374	0.0030	−0.0296	0.0108	0.0019
15	−0.0034	−0.0333	−0.0111	−0.0319	−0.0119	−0.0031
16	0.1524	0.0257	0.1562	−0.0084	0.1039	0.1353
17	0.1720	0.1865	0.1849	0.1230	0.0756	0.1723
18	−0.1231	−0.0054	−0.1230	0.0219	−0.1065	−0.1117
19	0.1022	0.0135	0.0933	−0.0180	0.0846	0.0936
20	0.1021	**0.2443**	0.0734	**0.1782**	−0.0461	0.1223
21	−0.0397	−0.0986	−0.0393	−0.0608	0.0041	−0.0455

Note: Components 1 and 6 relate to the nose width and the interocular distance.

Components 2 and 4 relate to the ratios of the mideye distance and the interocular distance to the nose width.

Components 3 and 5 relate to the ratios of the ear length and the lips-chin distance to the interocular distance.

**Table 6:** Parameters obtained from nonlinear regression for each component.

Component	*R*^*2*^	*α*	*r*	*σ*	*d*	*u*	*c*_*m*_
1	0.2887	0.2529	6.149	0.1252	2.430	−0.1281	6
2	0.2762	0.3013	−2.862	0.1493	2.662	−0.4609	3
3	0.2595	0.3533	1.531	0.1865	2.648	−0.3994	4
4	0.1780	0.0872	−8.263	0.0467	3.366	0.0531	3
5	0.1960	0.0789	7.296	0.0519	3.248	−0.0956	4
6	0.2571	0.2126	3.614	0.1179	2.857	0.1699	4
