# Supplementary material for: Brain responses to facial attractiveness induced by facial proportions: evidence from an fMRI study
Source: Sci Rep. 2016 Oct 25;6:35905. doi: 10.1038/srep35905 (PMC5078804; doi:10.1038/srep35905)
Supplement: Supplementary Information [file srep35905-s1.doc]

**Brain responses to facial attractiveness induced by facial proportions: evidence from an fMRI study**

*Supporting Information (SI)*

Hui Shen, Desmond K. P. Chau, Jianpo Su, Ling-Li Zeng, Weixiong Jiang,

Jufang He , Jintu Fan, Dewen Hu


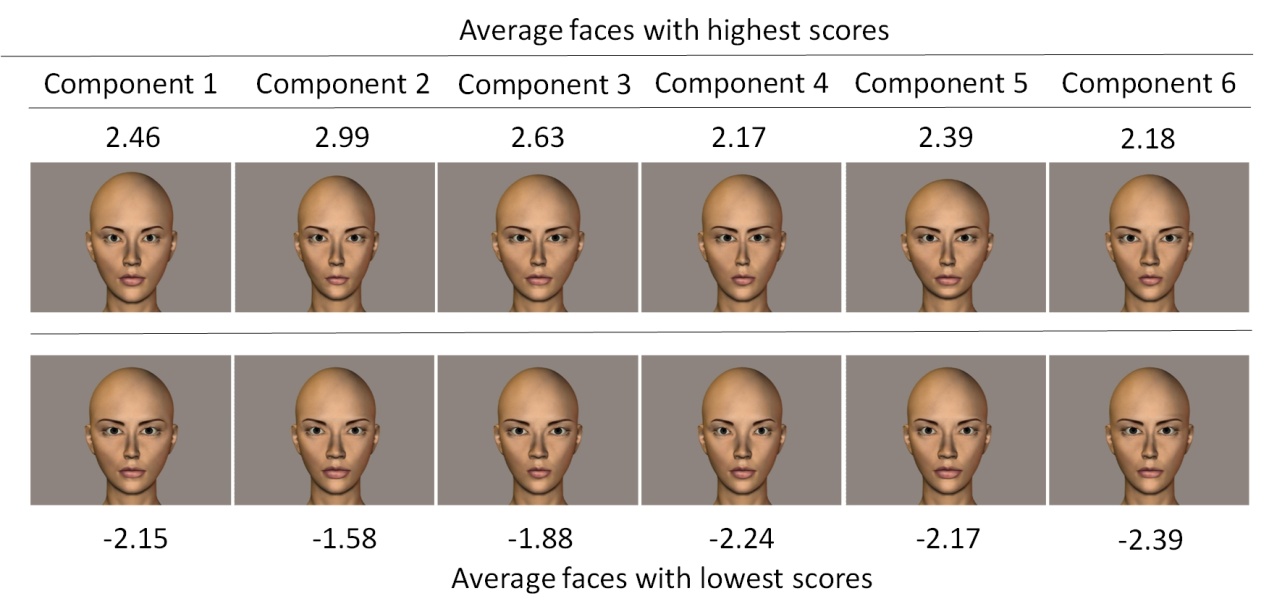


Figure S1. Effect of the six components of the face ratios identified with CCA on the facial images. For each component, the mean faces were obtained by averaging the 20 face images that had the highest or lowest scores of the component. The mean of these scores is shown below the face image.


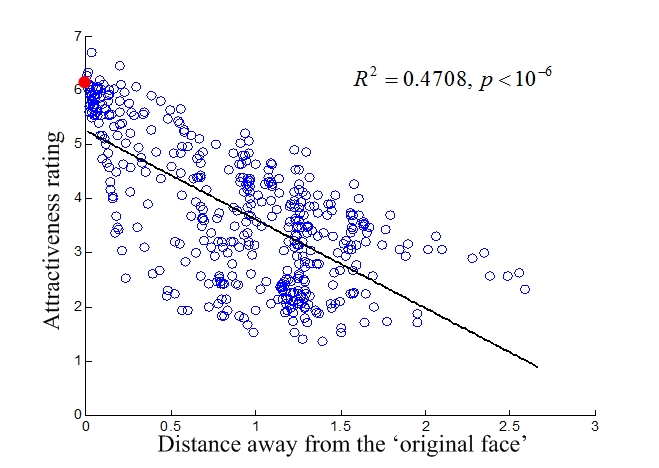


Figure S2. Attractiveness ratings of face images show significant negative correlation with the distances from the ‘original face’. In other words, a face image is observed as more attractive when its ratios are closer to the ‘original face’, which is highlighted by the red circle.
